# Male behavioural plasticity depends on maternal mating status in the two-spotted spider mite

**DOI:** 10.1007/s10493-017-0127-9

**Published:** 2017-04-21

**Authors:** Keiko Oku, Tom P. G. van den Beuken

**Affiliations:** 1 National Agriculture and Food Research Organization (NARO), Agricultural Research Center, Tsukuba, Japan; 2 Laboratory of Entomology, Wageningen University, P. O. Box 16, 6700 AA Wageningen, The Netherlands; 3 Institute for Biodiversity and Ecosystem Dynamics, University of Amsterdam, P. O. Box 94248, 1090 GE Amsterdam, The Netherlands

**Keywords:** Behavioural plasticity, Haplodiploid, Maternal mating status, Mate searching, *Tetranychus urticae*

## Abstract

In haplodiploid organisms including the two-spotted spider mite, *Tetranychus urticae* (Acari: Tetranychidae), both unmated and mated females can produce male offspring. A previous study reported that males produced by unmated females (UM males) find pre-reproductive females more quickly than males produced by mated females (M males) in *T. urticae*. However, it remains unclear what factors cause the difference. We investigated effects of maternal mating status on mate-searching behaviour of their sons by changing the sons’ developmental environment. In *T. urticae*, the primary sex ratio of mated-female colonies is female-biased. For both UM and M males, half of individuals were reared with males to imitate unmated-female colonies, whereas the rest were reared with females to imitate mated-female colonies. In UM males, individuals that had developed with males found pre-reproductive females more quickly than those that had developed with females. However, such a difference was not observed in M males. This indicates that behavioural response to the developmental environment differs between UM and M males. It means that the behavioural plasticity depends on maternal mating status. When males were individually reared, however, there was no significant difference in the mate-searching behaviour between UM and M males, indicating that maternal mating status does not independently affect their sons’ mate-searching behaviour. This study showed that male mate-searching behaviour is changed by their developmental environment and maternal mating status. This behavioural plasticity depending on maternal mating status is the first reported in haplodiploid organisms.

## Introduction

Phenotypes of organisms, such as behavioural and morphological traits, are plastic. A single genotype may produce different phenotypes in response to environmental conditions (West-Eberhard 1989). Thus, phenotypes are determined by genetic factors, environmental factors and the interactions between them (Falconer and Mackay 1996). Such plastic responses occur not only within one generation but also between generations. For example, plastic responses to conditions and/or environmental cues in maternal generations can be expressed as phenotypes in their offspring (Mousseau and Dingle 1991; Mousseau and Fox 1998; Marshall and Uller 2007). Thus, in order to determine what factors cause a phenotypic trait of organisms, it is important to consider effects not only within one generation but also between generations.

In haplodiploid organisms such as the two-spotted spider mite, *Tetranychus urticae* (Acari: Tetranychidae), haploid males develop from unfertilized eggs and diploid females develop from fertilized eggs (e.g., Helle and Bolland 1967; Heimpel and de Boer 2008). Thus, unmated females produce only unfertilized eggs (i.e., males), while mated females produce both unfertilized and fertilized eggs (i.e., males and females). Since both unmated and mated females produce male offspring, maternal mating status may affect male-offspring behavioural traits in haplodiploid organisms. To our knowledge, there are only two papers reporting effects of maternal mating status on male-offspring behaviour (the spider mite *T. urticae*: Ohzora and Yano 2008; the beetle *Coccotrypes dactyliperda*: Gottlieb et al. 2014). In both species, males produced by unmated females (hereafter called ‘UM males’) disperse at a higher proportion than males produced by mated females (‘M males’) (Ohzora and Yano 2008; Gottlieb et al. 2014). Moreover, in *T. urticae*, when one pre-reproductive female is displayed to both UM and M males at the same time, UM males find the female more quickly than M males (Ohzora and Yano 2008). In colonies established by unmated females, only the females (i.e. mothers) are available as mates, while there are many potential mates in colonies established by mated females. Hence, both studies deduced that different probabilities of encountering mates caused the behavioural differences between UM and M males (Ohzora and Yano 2008; Gottlieb et al. 2014). However, an alternative hypothesis that females modify male-offspring behaviour in response to their mating status remains to be investigated.

In several organisms, females modify offspring behavioural traits via eggs in response to their environmental conditions. For example, when mothers were exposed to predation risk, their offspring behave differently from individuals whose mothers were naïve (e.g., Giesing et al. 2011; Roche et al. 2012; McGhee et al. 2012; Bestion et al. 2014; Freinschlag and Schausberger 2016). Such behavioural plasticity may be adaptive and enhance offspring fitness (Bestion et al. 2014).

In this study, we focused on the mate-searching behaviour of *T. urticae* males and experimentally investigated the effects of maternal mating status on their sons’ behaviour by changing the environment in which the sons develop. If developmental environment determines male behaviour regardless of the mating status of their mothers, males that developed in colonies established by unmated females are expected to find females more quickly than those that developed in colonies established by mated females. By contrast, if maternal mating status determines the behaviour of male offspring, behavioural responses to the developmental environment are expected to differ between UM and M males. Here, we report male behavioural plasticity depending on maternal mating status in combination with developmental environment.

## Materials and methods

### Mites

**Fig. 1:**
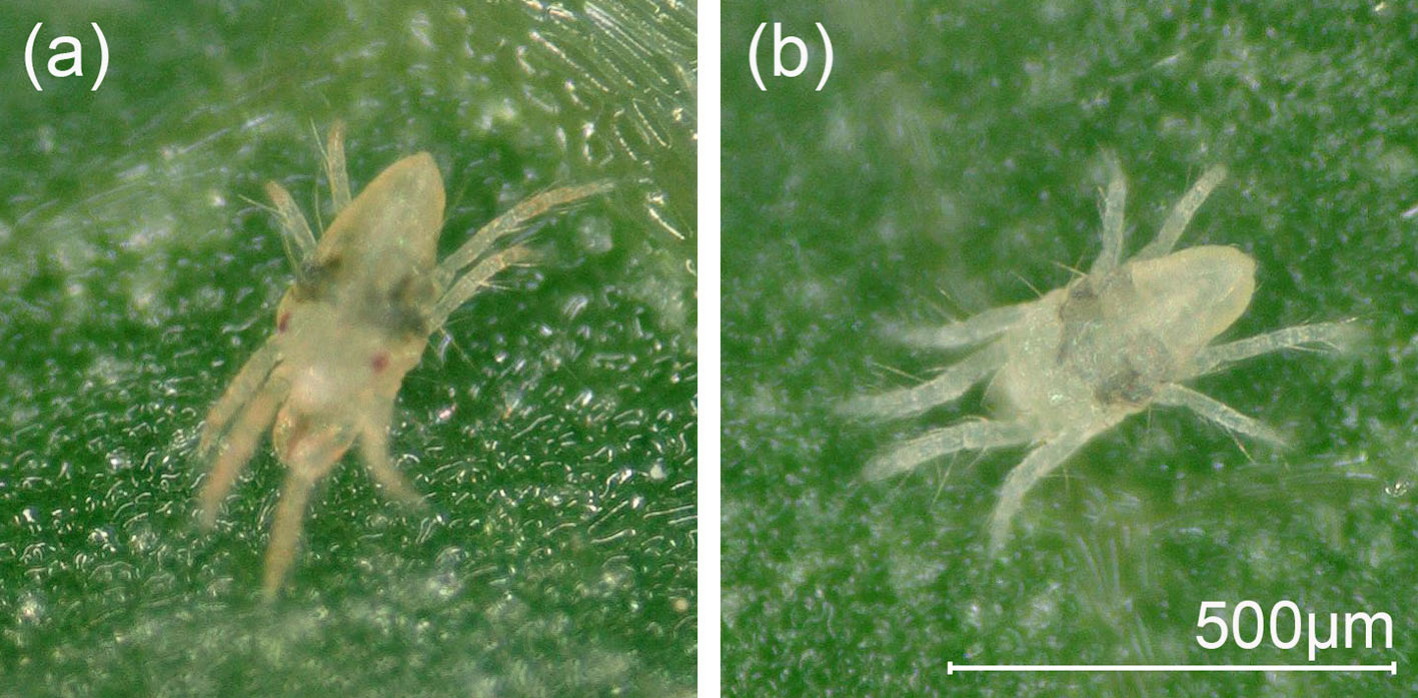
Adult male *Tetranychus urticae* **a** with and **b** without *red* eyespots

*Tetranychus urticae* was reared on expanded primary leaves of the kidney bean *Phaseolus vulgaris* (Leguminosae) placed onto water-saturated cotton wool in transparent plastic containers (10 cm diameter, 4 cm depth) in a climate room (25 ± 2 °C, 20–50% relative humidity, and L16:D8 photoperiod) at NARO Agricultural Research Center, Japan. The water-saturated cotton wool functioned to prevent mites from escaping from the leaves. A strain of *T. urticae* without red eyespots was established from one non-eyespot male (Fig. 1), which was accidentally found in the stock cultures, as follows: the non-eyespot male was allowed to mate with 20 unmated females arbitrarily selected from the stock cultures. All F1 individuals had eyespots. Thus, 20 F1 unmated females were transferred to clean leaf discs to obtain F2 males without eyespots. When F2 males without eyespots developed to the adult stage, they were allowed to mate with other F1 unmated females. Consequently, females without eyespots were obtained and they were reared for several generations. This strain of *T. urticae* without eyespots was used as an environmental factor for the first experiment (see details below).

The life cycle of *T. urticae* consists of egg, larva, quiescent larva, protonymph, quiescent protonymph, deutonymph, quiescent deutonymph and adult stages. To obtain unmated and mated females, 30–40 quiescent deutonymph females were arbitrarily selected from the stock cultures and transferred to 4–6 leaf discs placed on water-saturated cotton wool. Then, 15–20 adult males were added to half of the leaf discs for mating, whereas the others remained without mating. After 3 days, unmated and mated individual females were transferred to 1 × 1-cm leaf squares and allowed to oviposit for 1 day. Using this procedure, eggs were obtained for the following experiments. Unfertilized eggs produced by mated females are relatively smaller than fertilized eggs (Macke et al. 2011). To obtain unfertilized eggs produced by mated females as much as possible, we chose apparently smaller-sized eggs. Consequently, more than 50% of the eggs provided for the experiments were male (K. Oku, personal observation). Furthermore, to check whether mated females were indeed mated, they were removed from the leaf squares. Their eggs on the leaf squares were kept at 25 ± 2 °C and L16:D8 for 6 days. When offspring were only males, the mother was qualified as an unfertilized female and her offspring were excluded from experiments.

### Effects of developmental environment

Only for this experiment, both *T. urticae* individuals with and without red eyespots were used; the former was for observing male behaviour, whereas the latter was for controlling developmental environment. Colonies established by unmated females have only male offspring. By contrast, the primary sex ratio is female-biased in colonies established by mated females, which is around 0.75 (e.g., Wrensch and Young 1983; Krainacker and Carey 1990; Roeder 1992). To examine whether such developmental environment affects male mate-searching behaviour, three male eggs produced by either unmated or mated females with eyespots were transferred to 60 leaf squares (1 × 1 cm) and kept in the climate room for 5 days. For both males produced by unmated females (UM males) and males produced by mated females (M males), nine male juveniles (larvae and quiescent larvae) without eyespots were introduced to half of the leaf squares to imitate the environment of unmated-female colonies. Nine female juveniles without eyespots were introduced to the rest of the leaf squares to imitate the environment of mated-female colonies. Although we tried to pick proper individuals, it was difficult to distinguish between unfertilized and fertilized eggs or between male and female juveniles. Hence, the sex ratio varied among experimental arenas. The sex ratio, however, was extremely male-biased for males that had developed with males, whereas it was female-biased for males that had developed with females (K. Oku, personal observation). After 3–4 days, when males with eyespots developed to the quiescent deutonymph stage, they were transferred to new leaf squares where they were kept individually. After 2 days, half of UM and M males that had developed with males/females were marked with water-based pigment ink (a pen with a 0.05-mm point; Pilot, Tokyo, Japan). At the same time, deutonymph individual females with eyespots arbitrarily selected from the stock cultures were introduced to 7 × 7-mm leaf squares. Next day, the females had developed into quiescent deutonymphs. For each group (i.e., UM and M males), one male (2 days since adult emergence) that had developed in the presence of males and one male that had developed in the presence of females were introduced to the leaf squares with one female and observed for 30 min. We recorded which male first found the female. This experiment was repeated twice with 32–56 trials per replicate.

### Effects of maternal mating status

To examine whether maternal mating status affects male mate-searching behaviour independently, 144 eggs produced by 72 unmated females and 288 eggs produced by 72 mated females were transferred to—and kept individually on—1 × 1-cm leaf squares. The eggs were kept for 8–9 days in a climate room (25 ± 2 °C and L16:D8). When males developed to the quiescent deutonymph stage, they were individually transferred to new leaf squares. After 2 days, half of the UM and M males were marked with water-based pigment ink. At the same time, deutonymph females arbitrarily selected from stock cultures were transferred onto 7 × 7-mm leaf squares. On the next day, the females became quiescent deutonymphs. One UM male and one M male (2 days since adult emergence) were introduced onto the leaf squares with one female and observed for 30 min. We recorded which males first found the quiescent deutonymph females.

### Integrated effects of maternal mating status

To examine whether the *T. urticae* population used in this study shows that UM males find females more quickly than M males as reported by Ohzora and Yano (2008), eggs produced by unmated and mated females were prepared on leaf squares for each family in the same manner as described above. The eggs were kept for 8 days. When males developed to the quiescent deutonymph stage, they were separately transferred to clean leaf squares. After 2 days, half of the UM and M males were marked with water-based pigment ink. At the same time, deutonymph individual females were transferred to 7 × 7-mm leaf squares. Next day, the females became quiescent deutonymphs. One UM male and one M male (2 days since adult emergence) were introduced onto the leaf squares with one female and observed for 30 min. We recorded which males first found the quiescent deutonymph females. This experiment was repeated twice with 56 and 68 replicate trials, respectively.

### Statistical analysis

To determine whether male developmental environment affected their mate-searching behaviour for each male group (i.e., UM and M males) and whether maternal mating status affected male mate-searching behaviour, the data were analysed using replicated G tests (Sokal and Rohlf 1995). For the first and second experiments, because males that had developed to the quiescent stage at the 8th and 9th days were tested, we also considered day effects by calculating the heterogeneity. For the third experiment, males that had developed to the quiescent deutonymph stage only at the 8th day were tested. Thus, we calculated the heterogeneity only between replicates.

## Results and discussion

**Fig. 2:**
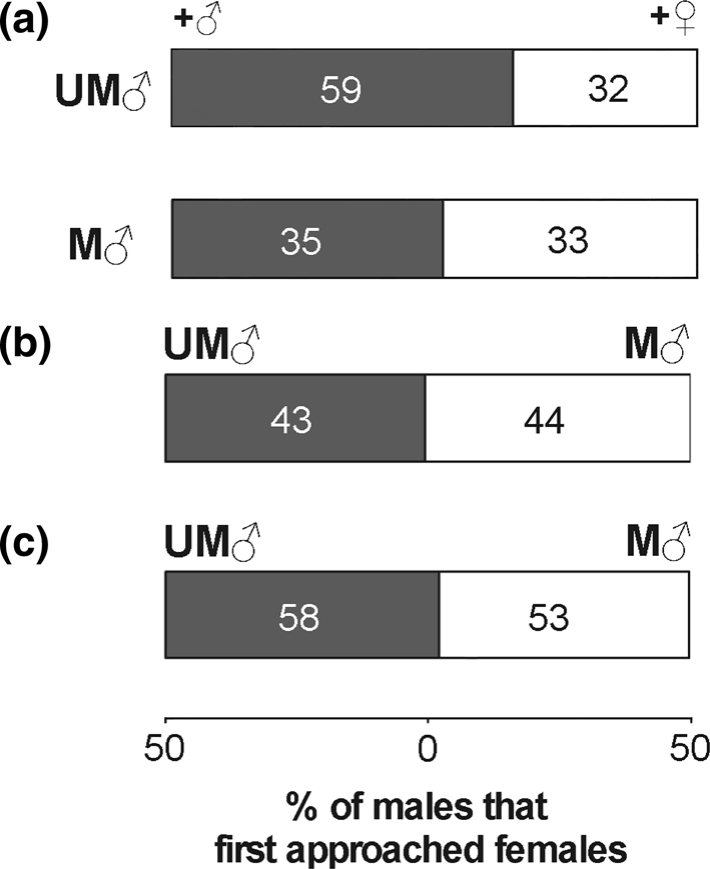
Pooled data from the comparison of mate-searching behaviour between males **a** that developed with males (+♂) and males that developed with females (+♀) for males produced by unmated females (UM♂) and males produced by mated females (M♂), respectively; **b** produced by unmated females (UM♂) and males produced by mated females (M♂) that developed individually; and **c** produced by unmated females (UM♂) and males produced by mated females (M♂) that developed with their family

No heterogeneity was detected among replicates in any of the experiments (Tables 1, 2, 3). Therefore, the data were pooled (Fig. 2) and further analysed per experiment. In UM males, individuals that had developed in the presence of males found females more quickly than those that had developed in the presence of immature females (Table 1; Fig. 2a). Such a difference, however, was not observed in M males (Table 1; Fig. 2a). This result indicates that behavioural responses to the developmental environment differed between UM and M males. It implies that this behavioural plasticity depends on maternal mating status.

**Table 1:** Results and statistical analysis of comparing mate-searching behaviour between males that developed with males (+♂) and males that developed with females (+♀) for males produced by unmated females (UM♂) and males produced by mated females (M♂), respectively

Types of ♂ and replicate no.	♂ that first found ♀			Individual *G* test			Replicated *G* test			
	+♂	+♀	Neither	*G*	*df*	*P*		*G*	*df*	*P*
UM♂										
1	21	11	4	3.178	1	0.075	Heterogeneity^a^	5.008	3	0.17
	3	2	1	0.201	1	0.65	Pooled^b^	8.133	1	0.004
2	17	15	0	0.125	1	0.72				
	18	4	2	9.636	1	0.002				
M♂										
1	12	10	0	0.182	1	0.67	Heterogeneity^a^	1.178	3	0.76
	4	6	0	0.403	1	0.53	Pooled^b^	0.059	1	0.81
2	10	11	6	0.048	1	0.83				
	9	6	1	0.604	1	0.44				

*Neither* means that any males did not approach females within the observation time of 30 min. Because males that had developed to the quiescent stage at the 8th and 9th days were tested separately, each replicate has two rows

^a^ *P* > 0.05 indicates that the frequency distributions of the replicates are not significantly different from each other

^b^ *P* < 0.05 indicates that the mate-searching behaviour differs between males that developed with males (+♂) and females (+♀)

**Table 2:** Results and statistical analysis of comparing mate-searching behaviour between males produced by unmated females (UM♂) and males produced by mated females (M♂) that developed individually

♂ that first found ♀			Individual *G* test			Replicated *G* test			
UM♂	M♂	Neither	*G*	*df*	*P*		*G*	*df*	*P*
21	25	5	0.348	1	0.56	Heterogeneity^a^	0.556	1	0.46
22	19	4	0.22	1	0.64	Pooled^b^	0.011	1	0.92

*Neither* means that any males did not approach females within the observation time of 30 min. Because males that had developed to the quiescent stage at the 8th and 9th days were tested separately, there are two rows

^a^ *P* > 0.05 indicates that the frequency distributions of the replicates are not significantly different from each other

^b^ *P* > 0.05 indicates that the mate-searching behaviour does not differ between UM♂ and M♂

**Table 3:** Results and statistical analysis of comparing mate-searching behaviour between males produced by unmated females (UM♂) and males produced by mated females (M♂) that developed with their family

Replicate no.	♂ that first found ♀			Individual *G* test			Replicated *G* test			
	UM♂	M♂	Neither	*G*	*df*	*P*		*G*	*df*	*P*
1	27	20	9	1.046	1	0.31	Heterogeneity^a^	0.442	1	0.51
2	31	33	4	0.063	1	0.80	Pooled^b^	0.225	1	0.64

*Neither* means that any males did not approach females within the observation time of 30 min

^a^ *P* > 0.05 indicates that the frequency distributions of the replicates are not significantly different from each other

^b^ *P* > 0.05 indicates that the mate-searching behaviour does not differ between UM♂ and M♂

When males were reared individually, however, there was no difference in the mate-searching behaviour between UM and M males (Table 2; Fig. 2b), indicating that maternal mating status does not independently affect the male behaviour. Unmated female *T. urticae* are likely to enhance mate-searching behaviour of male offspring, but it works in combination with developmental environments of the offspring. In *T. urticae*, adult females disperse and establish new colonies (Hussey and Parr 1963), and their offspring develop in the natal colony. When unmated females establish their own colonies, male offspring (i.e., UM males) develop with their brothers and have almost no opportunities to encounter mates there. Such a developmental environment can enhance UM males to behave to find females quickly. By contrast, when unmated females live with mated females, UM males have opportunities to encounter mates there. In such developmental environment, males do not need to alter their mate-searching behaviour. If males spend more time for mate searching, they will lose time for other activities, such as feeding. Thus, it might be costly for males to behave quickly for mate searching. On the other hand, whenever mated females established their own colonies, male offspring (i.e., M males) are destined to develop and mate with their sisters. Thus, this behavioural plasticity can be adaptive only for UM males that develop only with their brothers.

When males developed together with their own family, there was no difference in the mate-searching behaviour between UM and M males (Table 3; Fig. 2c). This result is inconsistent with that of Ohzora and Yano (2008). Although our design of experimental arenas was the same as that of Ohzora and Yano (2008), there were some differences in mite-rearing conditions. We individually transferred females to leaf squares, allowed them to oviposit for 1 day and removed them, whereas Ohzora and Yano (2008) transferred 15 females together to one leaf disc and allowed them to oviposit on the leaf disc until the end. When male offspring developed to the quiescent deutonymph stage, we separately transferred them to leaf squares, whereas Ohzora and Yano (2008) transferred them together to one leaf disc. These differences may have caused the inconsistence. Furthermore, although the sex ratio of the population used in Ohzora and Yano (2008) is unknown, the primary sex ratio (proportion of females) of the population used in the present study was 0.65 ± 0.03 (mean ± SE; n = 29) (K. Oku, unpubl. result). This sex ratio appears to be lower than the ca 0.75 shown in previous studies (Wrensch and Young 1983; Krainacker and Carey 1990; Roeder 1992). This difference could also be a factor to cause the inconsistence. Although several previous studies referred to Ohzora and Yano (2008) and used only UM males (Oku 2010, 2013; Oku and Saito 2014; Oku and Shimoda 2013; Sato et al. 2013), they did not confirm the behavioural difference between UM and M males in their own populations. It is necessary to clarify whether the behavioural difference in mate searching is universal among *T. urticae* populations. These points require further study.

The present study shows that male mate-searching behaviour is changed by developmental environment and maternal mating status. This behavioural plasticity depending on maternal mating status is the first report for haplodiploid organisms. Another plasticity in mating behaviour is also known in *T. urticae* and *T. kanzawai* males: mating behaviour alters in response to male density and/or their own age (Oku 2009; Sato et al. 2014). In these studies, however, only UM males were tested. This male behavioural plasticity may also depend on maternal mating status. When determining underlying mechanisms of phenotypic traits, it is important to consider factors not only within one generation but also between generations.
